# Complete Improvement of Severe Forearm Complex Regional Pain Syndrome with Six High-Dose Incobotulinumtoxin A Injections: Clinical Implications with Respect to the Literature

**DOI:** 10.3390/toxins16110488

**Published:** 2024-11-10

**Authors:** Harald Hefter, Marek Moll, Sara Samadzadeh

**Affiliations:** 1 Department of Neurology, University of Düsseldorf, Moorenstrasse 5, 40225 Düsseldorf, Germany; marek.moll@med.uni-duesseldorf.de (M.M.); sara.samadzadeh@yahoo.com (S.S.); 2 Charité—Universitätsmedizin Berlin, Corporate Member of Freie Universität Berlin and Humboldt-Universität zu Berlin, Experimental and Clinical Research Center, 13125 Berlin, Germany; 3 Department of Regional Health Research and Molecular Medicine, University of Southern Denmark, 5230 Odense, Denmark; 4 Department of Neurology, Slagelse Hospital, 4200 Slagelse, Denmark

**Keywords:** botulinum toxin, complex regional pain syndrome, dystonia, incobotulinum neurotoxin type A, low antigenicity

## Abstract

There is some evidence that injections of botulinum neurotoxin effectively reduce pain in complex regional pain syndromes (CRPSs). But no or little experience appears to exist for the application of incobotulinum neurotoxin type A (incoBoNT/A) in complex pain disorders. Here, a case of CRPS type I, characterized by severe symptoms in the left forearm is presented, showed significant continuous improvement following a series of six repetitive (painful) injections into the finger, hand, and forearm muscles of incoBoNT/A every 3 months, administered at declining doses varying between 500 and 100 U. Remarkably, this treatment regimen led to the complete resolution of pain, vaso- and sudomotor symptoms, and hand dystonia. This highlights the possible efficacy of incoBoNT/A in the treatment of CRPS and encourages the further exploration of incoBoNT/A’s role in the successful management of complex pain disorders.

## 1. Introduction

According to the International Association for the Study of Pain (IASP), complex regional pain syndrome (CRPS) is a complex primary pain disorder [1,2]. The diagnostic criteria of CRPS (of the IASP known as the Budapest criteria) are based on the assessment of clinical signs and symptoms [1]. CRPS is typically accompanied by sensory, vasomotor, sudomotor, and motor symptoms. Affected individuals experience debilitating burning pain, allodynia, and hyperpathia, coupled with vasomotor, sudomotor, and trophic changes [1,2]. In up to 20% of CRPS patients, additional dystonic muscle spasms will develop shortly after the onset of pain [3,4].

CRPS may be precipitated by a trauma or operation or can emerge spontaneously. Cases with a peripheral nerve injury (termed CRPS-II patients [5]) are distinguished from patients with an intact peripheral nervous system (referred to as CRPS-I patients [6]). Whether this distinction has clinical relevance is still a matter of research (see panel 2 in [2]).

CRPS primarily affects young adults, with typical manifestations in the leg or arm, and is often associated with specific HLA classes [7,8,9]. Historically, CRPS has been known by various names, including sympathetic reflex dystrophy and causalgia–dystonia syndrome [6], indicating the potential involvement of both the peripheral and central nervous systems (PNS and CNS, respectively) in the pathology of this multifaceted disorder. The possibility of CNS influence is further corroborated by successful treatments with deep brain stimulation (DBS) [10].

The pathophysiology of CRPS continues to be a subject of debate [2,11]. Theories range from a psychogenic origin [6] to a mismatch between the activity of peripheral efferent sympathetic and afferent sensory neurons causing sympathetic overflow [1,2,4,9,12,13,14]. Consequently, therapeutic approaches vary widely, from psychotherapy, physical and occupational therapy, pharmacotherapy (propranolol, calcitonin, bisphosphonates), and minimally invasive treatments such as sympathetic blocks, transcranial stimulation, and neuromodulation to DBS (for a review see [15]).

Successful CRPS treatment with botulinum neurotoxin A or B has been reported during the last 20 years [16,17,18]. Although controlled trials [19] and a meta-analysis [20] have been presented, the BoNT treatment of CRPS was not mentioned in a recent review [2]. The subcutaneous [21], intramuscular [22], and intraarticular [23] applications of BoNT/A have been executed with some success. The most common application of botulinum toxin (BoNT) in CRPS involves sympathetic blocks with BoNT injections (sometimes combined with local anesthetics) into the sympathetic ganglion [18,19,24] (for a recent review see [20]). The majority of these BoNT applications are performed with doses of abobotulinum neurotoxin type A (aboBoNT/A) of between 300 and 1000 U and onabotulinumneurotoxin type A (onaBoNT/A) doses of between 20 and 200 U ([20,25]). No treatment of CRPS by means of incobotulinum neurotoxin type A (incoBoNT/A) injections has been reported so far. Only a few reports on management of other pain syndromes by means of inBoNT/A injections have been presented [26,27,28]. But incoBoNT/A (Xeomin^®^) has the advantage that it has a low protein load, does not contain complex proteins, has a low antigenicity, has an antihidrotic effect, reduces dystonic and spastic muscular hyperactivity, and can safely be applied in doses of up to 800 U [29,30,31].

Furthermore, in most past reports on the BoNT treatment of CRPS, the effects of a single injection are described. In the present communication, the successful mitigation of a severe, typical forearm CRPS-I case achieved through a regimen of repeated intramuscular injections of high doses of incoBoNT/A is documented, ultimately leading to the complete resolution of all symptoms. This excellent outcome has several clinically relevant implications which will be highlighted after the case report.

## 2. Case Report

In this report, we present a case of a 33-year-old, right-handed male, previously a professional guitar player and currently employed in city administration, who began experiencing spontaneous burning pain in his left hand during the Christmas season. We have speculated that frequent guitar playing during that time triggered pain generation and the development of hand dystonia. The pain intensified progressively over the following days, rendering any tactile contact unbearably painful and leading to the cessation of his work duties.

Within two weeks, his hand and forearm had begun to swell, his skin color turned livid, and he developed tremulous and sporadic muscle jerks in his hand and forearm. Approximately four weeks after symptom onset, his fingers began to flex involuntarily and his hand deviated in the ulnar direction. Within another two weeks, his hand clenched completely shut, and any attempts at passive finger extension induced extreme pain.

Throughout the day, he found slight relief in leaning forward with his left arm hanging down, pressed between his legs. However, even in this position deemed most comfortable by him, his pain levels still hovered between 8 and 10 on an 11-point Likert scale. Despite several consultations with his primary care physician and subsequent treatment with anti-inflammatory drugs and opioids, his condition did not improve. Upon the appearance of the first dystonic symptoms, he was referred to two neurologists, including a movement disorder specialist at another German university hospital. Both diagnosed him with complex regional pain syndrome type I as no peripheral nervous system lesions could be detected by means of a detailed neurophysiological investigation, including needle EMG and nerve conduction velocity measurements.

Upon referral to the University of Düsseldorf’s pain management clinic, the patient fulfilled the Budapest criteria and the diagnosis of CRPS-I was confirmed again. A CT scan of his hand and forearm revealed osteopenia, edema, severe ulnar deviation, and flexed fingers. Despite the initiation of a treatment regimen with ß-blockers, calcitonin, pregabalin, and intensified opioid therapy, his hand and forearm swelling increased and the dystonia progressed. Further escalation led to his referral to our institution for BoNT injection therapy about 3 months after onset of symptoms.

**Figure 1:**
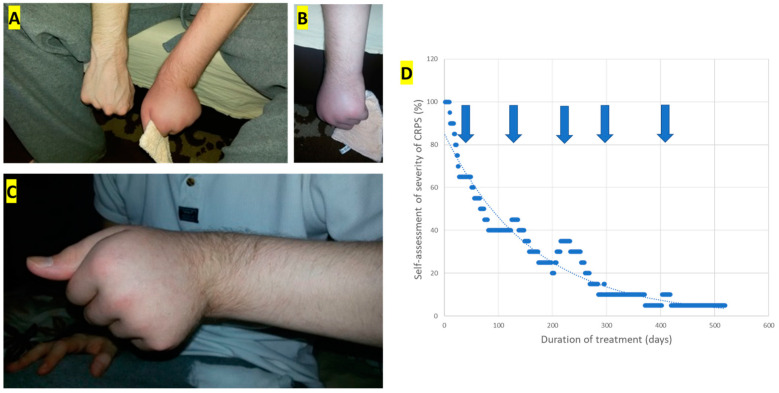
Photos were taken at the baseline visit: (**A**) Comparison of both hands; (**B**) livid skin color of the impaired left forearm; (**C**) edema and dystonia of the left forearm; (**D**) self-assessment of the severity of CRPS in % of the severity at the baseline visit (using a 21-point Lickert scale). The arrows indicate the application of injections 2 to 6.

Upon his first visit to our clinic, the patient appeared significantly debilitated, having lost more than 6 kg over the previous two months due to loss of appetite and inadequate sleep. His left hand and forearm were swollen and sweaty, the skin was of a livid hue, and he could not tolerate any skin contact (Figure 1A,B). His fist was clenched with a marked ulnar deviation (Figure 1C). Because of the hand dystonia and the severe pain, we decided to initiate high-dose BoNT/A therapy as soon as possible to influence hand dystonia, pain, and autonomic symptoms simultaneously.

The patient received his first injection the following day during a specialized workshop designed for treating patients with off-label indications. According to the clinical presentation, the muscles were injected, which contributed to the dystonia. The injected muscles included the adductor pollicis brevis, ulnar and radial hand flexors, and both the superficial and deep finger flexors. Also, injections of each spatium interosseum with 25 U incoBoNT/A were performed to influence the lumbricales and interossei muscles. Injections were performed without any guidance techniques by means of a 30 G needle of 4 cm length and fast skin penetrations to minimize the skin contact and duration of treatment. The total dose of incoBoNT/A administered was 500 U.

**Figure 2:**
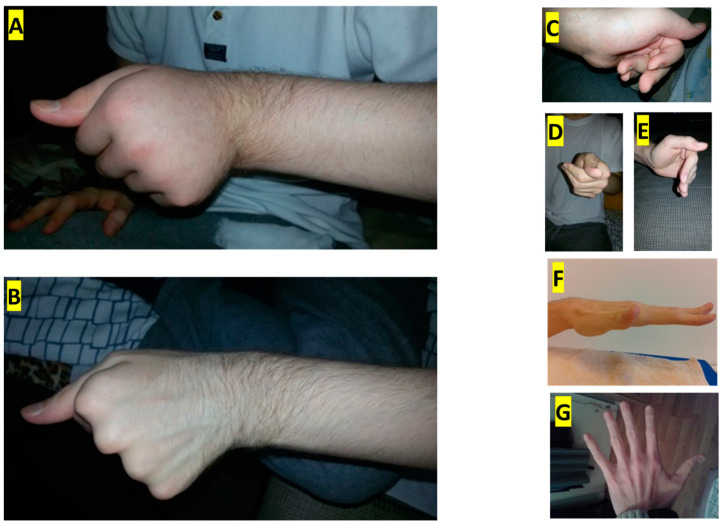
(**A**) Oedema and dystonia of the left hand and forearm before the 1st injection. (**B**) Improvement of oedema and dystonia of the left hand two weeks after 1st injection. Attempts to open the left hand voluntarily: (**C**) after the 1st injection, (**D**) after the 2nd injection, (**E**) after the 3rd injection, (**F**) after the 4th injection, and (**G**) after the 5th injection.

Remarkably, just a week after the initial injection, improvements in pain and swelling were observed (Figure 2B). Over the following weeks, the ulnar deviation gradually lessened, and the clenched fist began to relax (Figure 2D). Tremors and erratic muscle jerks ceased. For the first time since symptom onset, the patient was able to successfully attempt voluntary hand opening (Figure 2D).

The patient underwent a second treatment three months later, receiving another dose of 500 U incoBoNT/A. The improvements persisted, with the patient gaining increasing control over the opening of his hand (Figure 2E). To reduce paresis as a possible side-effect of intramuscular BoNT/A application, the subsequent two treatments, given at three-month intervals, involved the administration of 300 U each. After the fourth injection, the patient could briefly fully open his hand (Figure 2F). The final two treatments were conducted with 100 U each. Post five injections, the patient was able to keep his hand fully open for extended periods (Figure 2G, the ulnar deviation and swelling had subsided, and he regained individual finger mobility, allowing him to return to work. After the sixth injection, the patient even resumed playing the guitar. Remarkably, no relapse was observed in the following years (Table 1).

## 3. Clinically Relevant Implications

Because of the excellent outcome in the present case, we want to highlight the following four aspects:

(i) In contrast to the chronic CRPS patients being analyzed in the “meta-analysis of effectiveness and safety of botulinum toxin in the treatment of complex regional pain syndrome” [19] with a disease duration of between 2.2 and 11.8 years, the individual in the present case was injected about 4 months after the first onset of symptoms.
(ii) The patient was injected with much higher doses than usually used for BoNT treatment of CRPS.
(iii) The injections were performed every three months, well before the effect of the previous injection had declined, and were placed where the patient had the most pain and muscular hyperactivity.
(iv) incoBoNT/A was used because of its low antigenicity, which is important when repeated injections with high doses are performed.

In summary, the approach of injecting CRPS cases soon after onset of symptoms, directly at the location of maximal complaints with high doses of incoBoNT/A every 3 months seems to be a promising management of CRPS symptoms; this of course has to be confirmed by controlled trials.

## 4. Discussion

Botulinum neurotoxins have demonstrated success in treating various pain syndromes, including central neuropathic pain, chronic musculoskeletal disorders, post-herpetic neuralgia, migraines, and trigeminal neuralgia, where the blocking of the release of CGRP and/or substance P or other neuroinflammatory agents is hypothesized to be the underlying mechanism [25,28,32,33,34]. Their versatile utility also extends to reducing glandular hyperactivities such as excessive sweating and drooling [31], as well as controlling muscular hyperactivity by inhibiting the release of acetylcholine at the neuromuscular endplate [27,35]. These combined attributes suggest botulinum neurotoxins’ potential in treating the vasomotor, sudomotor, and motor symptoms inherent in CRPS.

Prior reports affirm the success of treating CRPS with botulinum neurotoxin A or B [18,20]. Most patients showed partial improvements following intradermal, intramuscular, and intraarticular applications of BoNT/A [21,22,23]. These partial improvements could be sustained in some patients through repetitive BoNT injections, while others experienced continued CRPS progression despite symptomatic relief. The most frequent application of BoNT in CRPS are sympathetic blocks created by BoNT injections into the sympathetic ganglion [18,19,24]. Typically, these blocks are performed with doses of 75 to 200 U OnabotulinumtoxinA (onaBoNT/A) [20,25], significantly lower than the 500 U of incoBoNT/A employed in our study.

Given the activated and alert state of the immune system in CRPS patients, capable of detecting even low doses of botulinum neurotoxin as demonstrated in long-term treated patients with cervical dystonia [36,37], the use of low doses in the treatment of CRPS is a reasonable approach. However, because of the known significantly lower antigenicity of incoBoNT/A compared to abo- or onaBoNT/A [29], we decided to use this BoNT/A-preparation in our study, starting with an initial dose of 500 U, which was well below the 800 U tested in the TOWER-study [30] but well above the so far used doses of onaBoNT/A for the treatment of CRPS. It should be noted that neither abo-, ona-, nor incoBoNT/A, nor timabotulinum neurotoxin type B (rimaBoNT/B), are licensed for the treatment of CRPS.

Furthermore, there are no recommendations regarding appropriate doses for CRPS treatment. With BoNT/A’s demonstrated reduction in sweating and muscular hyperactivity, we decided to inject high doses, hypothesizing that diffusion could potentially reach the neighboring tissues of hand and forearm muscles. Interestingly, the effects on sudomotor and vasomotor symptoms appeared more rapidly than those on motor symptoms.

At onset of incoBoNT/A therapy paresis as a possible side-effect of intramuscular BoNT injections was irrelevant since the patient could not use his hand anyway. However, with the improvement of hand control, the total dose of incoBoNT/A was reduced step by step to avoid finger and hand muscle weakness.

So far, only CRPS patients with dystonia are injected with BoNT [20]. Therefore, the question remains whether CRPS patients without dystonia can be equally well treated with BoNT. Despite the positive outcome in the present case, a single “golden responder”, is encouraging, we must remain cautious in extending this observation too far. Nonetheless, this case inspires further investigation into the potential benefit of treating additional CRPS cases with repetitive high doses of incoBoNT/A.

## 5. Conclusions and Recommendation

This short communication suggests that even severe instances of CRPS can be effectively managed with targeted intramuscular injections of incoBoNT/A. Notably, since CRPS frequently exhibits localized onset, we propose the early implementation of high-dose incoBoNT/A treatment immediately following a confirmed diagnosis of CRPS. This approach could potentially prevent further disease progression and provide patients with the possibility of achieving complete symptom resolution.

## Figures and Tables

**Table 1:** Dose and dose distribution of the six incobotulinumtoxin A injections.

Number of Injection	Dose (U) of incoBoNT/A	Injection Sites
1	500	2 × 75 U per ulnar hand flexors 2 × 25 U per radial hand flexors 2 × 50 U per superficial finger flexors 2 × 50 U per deep finger flexors 100 U to 25 U per spatium interosseum injected from dorsal direction
2	500	Injection 2 was applicated exactly as injection 1
3	300	2 × 50 U per ulnar hand flexors 2 × 25 U per superficial finger flexors 2 × 25 U per deep finger flexors 100 U to 25 U per spatium interosseum injected from dorsal direction
4	300	Injection 4 was applicated exactly as injection 3
5	100	100 U to 25 U per spatium interosseum injected from dorsal direction
6	100	Injection 5 was applicated exactly as injection 6

## Data Availability

The data presented in this study are available on request from the corresponding author due to the restrictions of privacy and ethics.
